# Eye-tracking evidence shows that non-fit messaging impacts attention, attitudes and choice

**DOI:** 10.1371/journal.pone.0205993

**Published:** 2018-10-26

**Authors:** Ilona Fridman, Peter A. Ubel, E. Tory Higgins

**Affiliations:** 1 Management Department, Columbia Business School, New York, New York, United States of America; 2 Fuqua School of Business School, Sanford School of Public Policy, Duke University, Durham, North Carolina, United States of America; Technion Israel Institute of Technology, ISRAEL

## Abstract

When patients have strong initial attitudes about a medical intervention, they might not be open to learning new information when choosing whether or not to receive the intervention. We aim to show that non-fit messaging (messages framed in a manner that is incongruent with recipients’ motivational orientation) can increase attention to the message content, thereby de-intensifying an initial attitude bias and reducing the influence of this bias on choice. In this study, 196 students received information about the pros and cons of a vaccine, framed in either a fit or non-fit manner with their motivational orientation. The results show that when information was presented in a non-fit (*vs*. fit) manner, the strength of participants’ initial attitude was reduced. An eye-tracking procedure indicated that participants read information more thoroughly (measured by the average length of fixation time while reading) in the non-fit condition versus fit condition. This average time of fixation mediated the effect of message framing on the strength of people’s attitudes. A reduction in attitude was associated with participants’ ability to recall the given information correctly and make a choice consistent with the provided information. Non-fit messaging increases individuals’ willingness to process information when individuals’ pre-existing attitude biases might otherwise cause them to make uninformed decisions.

## Introduction

Imagine that you have a generally positive attitude toward a type of medical procedure. What is the likelihood that you would carefully examine information about it before agreeing to take it? Studies have shown that people tend to process information rapidly and find it unconvincing if it contradicts their initial attitudes [1]. Therefore, strong initial positive attitudes toward a medical procedure might prevent you from exploring the potential risks of that procedure. This, in turn, might result in an uninformed decision, such as choosing to undergo the procedure without fully appreciating its risks.

When facing important decisions, people might benefit from interventions that overcome their tendency to make hasty choices that align with their initial attitude bias. A recent study has shown that non-fit messaging (i.e., framing a message to be incongruent with the recipient’s motivational orientation) was associated with the reduction of initial strong attitudes [2]. In the current study, we utilize regulatory focus and fit theories to examine the effects from individuals receiving regulatory non-fit (*vs*. fit) messages, i.e., receiving a message that emphasizes the harms of an option when their motivational orientation is to consider the benefits of an option or receiving a message that emphasizes the benefits of an option when their motivational orientation is to consider the harms of an option. We explore the effect of such non-fit message framing on attitude change in the context of a hypothetical decision. Utilizing eye-tracking equipment, we examine: a) what information individuals attend to and how thoroughly they read the message for a non-fit (*vs*. fit) message; b) whether more thorough information processing is associated with a change in attitude and subsequent choice. This experimental design and eye-tracking procedure allow us to empirically test theoretical assumptions about non-fit messages and provide insight on how such message framing could be utilized to support informed decisions.

## Regulatory focus and regulatory fit

### Regulatory focus

According to regulatory focus theory, many decisions are influenced by individuals’ tendency to focus on achieving benefits (promotion motivational focus) or avoiding harms (prevention motivational focus) [3, 4]. With promotion focus, individuals are concerned about growth, accomplishments, and achieving better states. When making a decision, they consider what they could gain by choosing an option X. In contrast, with prevention focus, individuals are concerned about safety, security, and maintaining a satisfactory status quo against a worse, negative state. When making a decision, they want to know how an option X could stop losses.

Whether a person is more promotion- or prevention-oriented depends on both personal and situational characteristics. Individuals’ chronic orientations are influenced in part by whether they grew up in a social environment that emphasized accomplishments and growth (promotion) or one that emphasized safety and obligations (prevention) [5]. Strongly defined situations can also influence whether individuals approach a decision with a promotion or prevention focus [6]. For example, a decision about healing a current injury could induce a promotion focus, with individuals considering how treatment could help them promote healing that brings them to a better state. In contrast, a decision about an elective surgery could induce a prevention focus, with individuals considering how treatment could help prevent a worsening of their current state. No matter the reason for a person’s regulatory focus, promotion and prevention foci influence the type of information they attend to, how they interpret this information, and what they evaluate as worth acting upon when making decisions [7, 8].

### Regulatory fit and non-fit

Individuals can operate in a decision-making context that either matches or supports their current motivational orientation (a fit) or mismatches or disrupts their current motivational orientation (a non-fit). Promotion-focused individuals experience regulatory fit when they operate in a context that emphasizes growth and gains, but experience non-fit when the context emphasizes safety and losses. In contrast, prevention-focused individuals experience regulatory fit when they operate in a context that emphasizes safety and losses, but experience non-fit when the context emphasizes growth and gains [9, 10].

Regulatory fit has been shown to intensify various outcomes, including attitudes, evaluations, willingness to buy a product, or engage in healthy behaviors [11–16]. For example, while making a medical decision, individuals might ask, “how can I improve my health?”, or “how can I stop disease?” The first question would create regulatory fit for promotion-focused individuals, whereas the second question would create regulatory fit for prevention-focused individuals. When individuals experience regulatory fit, they “feel right” and are more confident in their initial attitudes [9].

In contrast to regulatory fit, regulatory non-fit has a de-intensifying effect on individuals’ initial attitudes and evaluations. This de-intensification effect of regulatory non-fit results from individuals’ making decisions in a manner that disrupts their motivational orientation [9]. For example, a disruption would occur when individuals who are initially concerned about the benefits of treatment (promotion focus) receive information that emphasizes how this treatment helps to stop losses or, alternatively, when individuals who are initially concerned about safety and stopping losses (prevention focus) receive information about the benefits (gains) of the treatment in improving health. When individuals experience regulatory non-fit, they “feel wrong” and are less confident in their initial attitudes [16], which de-intensifies their initial attitude.

#### Regulatory non-fit and motivation to process information

Previous research has demonstrated that regulatory non-fit motivates individuals to process information more thoroughly [17, 18]. In these studies, while experiencing non-fit (*vs*. fit), participants were more likely to make accurate evaluations in the presence of misleading heuristics. Researchers suggested that participants made better evaluations because they switched from superficial to more thorough message processing if they experienced regulatory non-fit (*vs*. fit). These findings are consistent with previous work investigating how incongruent messages (in various respects) facilitate elaborative thinking [19–21]. In this study, we used eye-tracking software and equipment to observe how individuals read information when it is framed in a fit or non-fit manner with their motivational focus.

Previous research shows that individuals tend to have longer eye fixation–eyes stopped and hold central vision–while they engage in information processing [22–24]. For instance, individuals fixate longer when they encountered an infrequent word or a misspelled word [25, 26].

We hypothesize that in the non-fit condition, participants will have longer fixations processing information more thoroughly than in the fit condition.

Hypothesis 1: Participants’ fixation duration will be longer in the regulatory non-fit condition than in the fit condition.

#### Regulatory non-fit and attitude change

While past research indicates that increased motivation to process a persuasive message does not always lead to attitude change [27, 28], framing information in a regulatory non-fit manner has been associated with both increased information processing [17] and attitude change [2].

In our previous research, we found that if a message created a non-fit (*vs*. fit) experience, it reduced (de-intensified) participants’ initial negative attitudes towards potentially beneficial options [2]. In that research, participants imagined having advanced cancer and receiving advice to discontinue cancer treatment. The advice was framed to create either a regulatory fit or non-fit experience for participants. Participants who initially disliked the recommended option disliked it *less* after they received advice that created a non-fit (*vs*. fit) experience; that is, the non-fit de-intensified their initial negative attitude. Participants were also more willing to follow the advice.

The findings from that research suggest that regulatory non-fit, when coupled with counter-attitudinal arguments (in that case’ advice), increases the likelihood that individuals will change their initial attitude.

At the same time, in the context of medical decision-making, patients frequently receive information that is both pro- and counter-attitudinal for them. This led us to ask: could regulatory non-fit be helpful in reducing initial attitudes when participants encounter both pro- and counter-attitudinal information?

In this study, we provided both pro- and counter-attitudinal information to participants aiming to explore: a) what information individuals pay attention to, and b) how thoroughly they process it in the non-fit condition versus the fit condition. This approach will allow us to explore whether a regulatory non-fit experience leads to attitude change if both pro- and counter- information is presented, extending the regulatory non-fit theory by describing how the relation between the message content and framing (fit *vs*. non-fit) influences attitude change.

Hypothesis 2 (exploratory): Regulatory non-fit condition will enhance participants’ motivation to read pros, cons, or both.
Hypothesis 3: Participants will reduce the intensity of initial attitudes in the non-fit condition more than in the fit condition.
Hypothesis 4: Increased information processing in the regulatory non-fit condition will lead to reducing the strength of participants’ initial attitude.

#### Regulatory non-fit and decision making

If participants do change their initial attitudes after experiencing regulatory non-fit and receiving pro- and counter-attitudinal information, does it help them make a better decision? To test this question, we created a vignette about a hypothetical harmful vaccine. In our study, the statistics about the fictitious vaccine suggests that it causes severe side effects more often than it protects from disease.

We recruited students, expecting that most would have positive attitudes toward vaccination. Strong positive attitudes toward vaccination might prevent participants from exploring the information about a new (fictitious) vaccine. This population and the context allowed us to experiment with naturally occurring attitudes. Although it would be rare that a positive attitude toward vaccination would need to be reduced, more generally there are times when a positive attitude toward a medical procedure can be problematic, as when individuals have positive attitudes toward invasive procedures that provide little or no benefit for patients (e.g., surgery for early stage of prostate cancer or early stage thyroid cancer).

The question we aim to answer in this experiment is whether participants with a strong positive attitude toward a procedure—in this case, a (fictitious) vaccination—sufficiently consider its’ poor risk/benefit ratio when making their decision? According to attention selectivity bias [29], individuals might be less motivated to invest cognitive efforts in processing the information that is counter-attitudinal for them. Favoring vaccination, our participants, due to their attention selectivity bias, might pay superficial attention to the statistics that suggest negative effects of this particular vaccine. Consequently, they might choose to vaccinate despite the undue risk of the (fictitious) vaccine. Information about pros and cons framed in a regulatory non-fit manner might mitigate initial positive attitudes and, therefore, reduce information selectivity bias. Thus, they would then pay more attention to the risks of the vaccine. This, in turn, could motivate them to choose *not* to vaccinate. We aim to show that regulatory non-fit and its effect on de-intensifying a prior attitude can be helpful in facilitating informed decisions.

Hypothesis 5: Participants will be less likely to take the fictitious harmful vaccine in the regulatory non-fit condition than in the regulatory fit condition.
Hypothesis 6: Reduction of initial positive attitudes in the regulatory non-fit condition will increase the likelihood that participants will choose not to take the fictitious harmful vaccine.
Hypothesis 7: Participants will be more likely to re-call statistics about the fictitious harmful vaccine in the regulatory non-fit condition than in the regulatory fit condition.
Hypothesis 8: Reduction of initial positive attitudes in the regulatory non-fit condition will increase the likelihood that participants will correctly re-call statistics about the fictitious harmful vaccine.

## Methods

### Participants

A total of 201 students were recruited for this experiment. Five participants were excluded for failing to complete the study. The resulting sample included 196 participants. The following demographics were reported: average age was 26 (*SD* = 7); 60% of participants were female students; 45% Asian, 32% White, 11% Black, 13% Others. This study was approved by Columbia Morning Site IRB.

### Design & procedure

Students were recruited through the SONA website, which provides information to graduate and undergraduate students about behavioral research. Participants chose convenient time slots and came to a behavioral laboratory to complete this study for monetary compensation.

Upon arriving at the laboratory, participants read an electronic consent form. The consent summarized the study procedure and informed participants that they were free to quit the study at any time without a penalty. Participants received the contact information of the researchers and IRB office for any questions that might arise after the study. Participants then were informed that by clicking the button “proceed to the study” they agree to the terms of the consent form. All the participants who agreed to participate received the monetary compensation for their time. The consent procedure was approved by Columbia Morning Site IRB.

**Fig 1:**
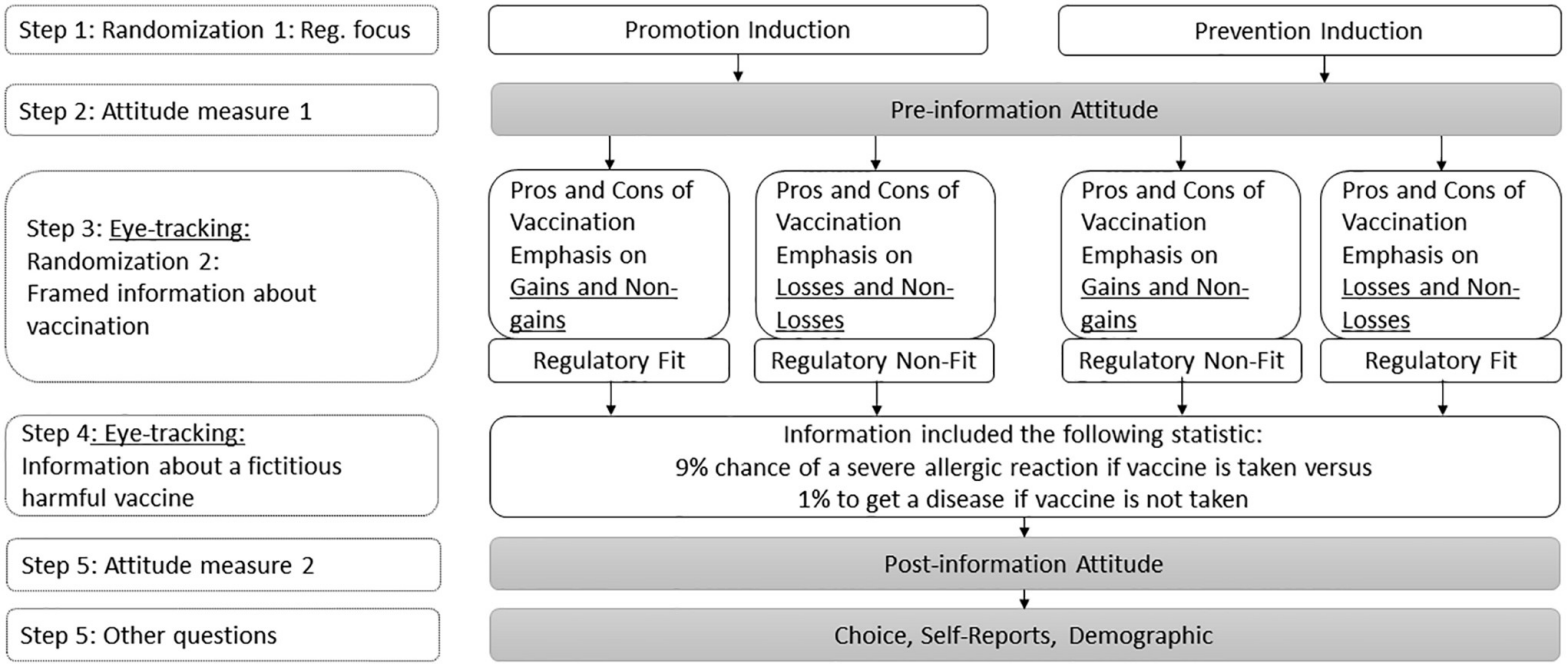
Study design.

A step-by-step illustration of the study design is outlined in Fig 1. In the first step, participants were primed to approach decisions with either a promotion or prevention focus. Following a validated procedure [8], we induced promotion focus by asking participants to write about three instances when they successfully achieved gains. To induce prevention focus, we asked participants to write about three instances when they successfully avoided losses. For the instructions, see Supplementary materials S1 Text.

In the second step, participants reported their general initial attitudes toward vaccination by providing their agreement with six statements about vaccination.

In the third step, participants imagined that they were visiting a doctor before traveling to a country expecting a dangerous outbreak of “Mepharagic” fever. The doctor provided two pages of information. The first page included information about the general pros and cons of vaccination. The information about pros and cons was framed either in terms of gains or losses. To emphasize gains, the message described how vaccination can help individuals to achieve better states (pros) or fail in helping individuals to achieve better states (cons). To emphasize losses, the message described how vaccination can help avoid losses (pros) or fail to help individuals avoid losses (cons). When combined with the priming of either a promotion or prevention focus from the first step, this framing of the message information in terms of gains and losses induced fit or non-fit experiences.

In the fourth step, participants received information about a (fictitious) vaccine that harmed more people than it helped. The information about the fictitious vaccine was identical for all participants across all conditions. Participants learned that there was only a 1% chance of contracting the disease if they chose to travel without taking the vaccine. However, there was a 9% chance of experiencing a severe allergic reaction from the vaccine, which was worse than the disease itself. Participants with positive prior attitudes toward vaccination might read the statistics about the negative effects of this particular vaccine superficially and choose to take it anyhow. However, regulatory non-fit could reduce (i.e., de-intensify) positive attitudes towards vaccination, making it less likely that these participants’ would choose to receive the harmful vaccine (with choice being measured in step six).

In the fifth step, participants once again reported their general attitudes toward vaccination. The items were identical to the items in step 2.

Finally, in step six, participants chose whether or not to get the (fictitious vaccine), and reflected on their experience in the experiment by providing self-reports on various measures. Additionally, they answered demographic questions, reported whether they had glasses, contact lenses, any eye conditions, and whether they had ever refused to take a vaccine for themselves.

### Measures

#### Regulatory fit and non-fit conditions

Those participants who completed the promotion orientation induction experienced regulatory fit if they read message information that emphasized gains of vaccination; they experienced non-fit if they read information that emphasized losses of vaccination. In contrast, those participants who completed the prevention orientation induction experienced regulatory fit if they read message information that emphasized losses; they experienced non-fit if they read information that emphasized gains of vaccination.

#### Eye-tracking data

Participants’ eye movements were recorded by Tobii equipment and processed by Tobii software. Recording resolution of Tobii TX-300 Eye Tracker was 1920 x 1080 (11.5" x 20"), with I-VT filter for stationary eye-tracker applied.

**Fig 2:**
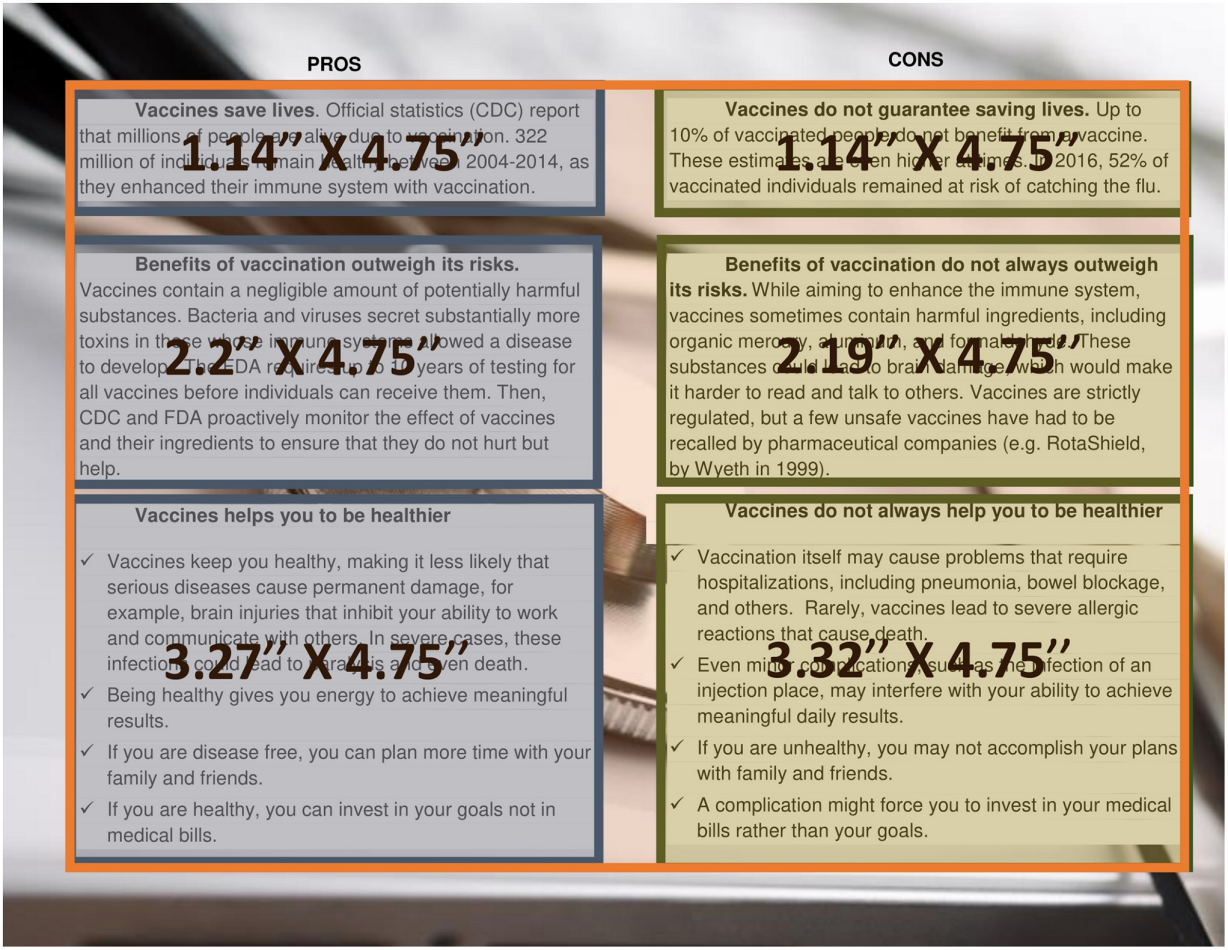
Areas of interest: Pros & cons (left) and information about vaccine (right).

Our stimuli materials consisted of a screen page about the general pros and cons of vaccines (11.5” x 8.5”) and a screen page about a fictitious vaccine (8.5” x 11.00”). To collect eye-tracking data, we highlighted the areas in our stimuli materials prior the experiment. The layout of these areas is presented in Fig 2. In the analysis, we used a summary statistic from 3 pros (variable “Pros”) and 3 cons (variable “Cons”) area of interest. Additionally, we used an area of interests (orange highlight) that covered the whole text on the page Pros & Cons (variable Pros & Cons).

Both the total number and the duration of fixations were recorded for each area of interest. Aiming to collect a precise measurement of eye-tracking behavior, we followed default settings of Tobii Soft that suggest 60ms as a conservative threshold for fixation duration that accounts for complex oculomotor behavior such as express saccades (Tobii Manual, p. 56). “Fixations are those times when our eyes essentially stop scanning about the scene, holding the central foveal vision in place so that the visual system can take in detailed information about what is being looked at” (Tobii Pro Spectrum Eye Tracker User’s Manual, 2018). The average fixation duration for reading English is ~200-250ms [30]. Consistent with this statistic, the average fixation duration (AFD) ranged between 210–220ms in this study.

Additionally, we evaluated total duration fixation (TFD). It is the summary of all fixations in the area of the interest. Following previous research [22], we accounted for individual differences in fixation time by dividing total duration time within each area of interest on total duration time across the whole document. This measure does not depend on the number of fixations and allows us to account for participants’ strategy of information search while reading. For example, if a participant skimmed the text, the participant might have large average fixations but might have a relatively small total duration of fixations. While average duration fixation is a motivation to process information more thoroughly, total duration fixation is a proxy of participants’ motivation to explore more details in the text.

Although, TFD and AFD are related, correlations coefficients were relatively small: Pros&Cons TFD vs. AFD *r*. = .160, *p* = .03; Vaccination information TFD vs. AFD. *r*. = .24, *p* = .001, for the full correlation table see supplementary materials, S1 Table.

#### Attitudes toward vaccinations

Participants reported to what extent they agreed with six statements about vaccinations. These included statements such as “Vaccines are effective in preventing diseases” or “Whenever it is possible, I choose to avoid getting vaccinated (reversed item).” Four statements reflected positive beliefs about vaccination, while two statements reflected negative beliefs about vaccination. The list of questions is included in supplementary documents S1 Text. Participants provided their agreement with the same six items before they read the framed information about vaccination and after they completed reading the framed and unframed information. Participants’ responses were averaged (with the two negative items reversed) to create a measure of positive vaccination attitude: pre-information positive attitude (Cronbach’s α = 0.79) and post-information positive attitude (Cronbach’s α = 0.81). We followed theoretical suggestions in statistical analysis in evaluating the level of reliability of the attitude measure. Acceptable values of Cronbach’s α ranges from 0.70 to 95 [31–33]. Our Cronbach alfa’s for both pre- and post- measures are in the middle range of the acceptance.

In mediation analyses, we used attitude change as a variable. It was calculated by subtracting pre-attitude from post-attitude. In this attitude change variable, more negative (lower) numbers indicated stronger attitude change (i.e., reduction of positive attitude).

#### Choice and self-reflection of information processing

Upon reading information and reporting attitudes, participants chose whether or not to get the (fictitious) harmful vaccine. The answers were coded as the following: “0” = no (reject); “1” = yes (accept). We asked participants to report the extent to which they found the information about vaccination convincing. They also reported how satisfied they were with the information provided, and to what extent they trusted in their physician and vaccination. Additionally, participants recalled the provided risks and benefits of the particular vaccine by answering two questions: (1) “Could you please provide a percentage estimate of what your chances are to get mepharagic fever if you visit Buracao?” and (2) “Could you please provide a percentage estimate of what your chances are to experience an allergic reaction if you get the vaccine?” The correct answer to *both* questions was coded as “1”. If participants made a mistake answering either one of these questions, their answer was coded as “0”.

#### Covariates

Participants reported whether they had contact lenses or glasses as well as whether they had any medical condition that might affect the eye-tracker performance (no one had a medical condition). Participants also reported their actual decisions about past vaccinations. Specifically, we asked if they had ever refused to have a vaccination (18% disclosed that they had refused being vaccinated at least once).

## Results

### Regulatory non-fit and motivation to process information (Eye-tracking data)

We compared participants’ average fixation duration (AFD) between the fit and non-fit conditions, to test our central hypothesis predicting that regulatory non-fit experience increases how long individuals fixate while reading. We compared AFD of pros, cons, and information about the vaccine among participants between the fit and non-fit conditions, using MANOVA test to account for multiple comparisons. MANOVA revealed significant results, *F*(3, 177) = 4.08, *p* = .01, η^2^_partial_ = .07; with covariates, such as contact lenses/glasses and past experiences with vaccination the result remains significant (*p* = .01). Individual results for each variable are presented in Table 1.

**Table 1:** Comparison of average duration fixation between fit and non-fit conditions.

Areas of interest	Non-Fit, *M*(SD)	Fit, *M*(SD)	F	*P*	N*	η^2^_partial_
Pros	0.22 (0.03)	0.21(0.03)	10.35	.002	181	.55
Cons	0.22 (0.03)	0.21(0.04)	3.98	.05	181	.22
Vaccine Info	0.22 (0.03)	0.21(0.03)	2.41	.12	181	.13

Average fixation duration is recorded in seconds.

* Sample size is reduced because some participants did not fixate in all 3 pros or all 3 cons areas of interest.

While we expected and found that participants read information more thoroughly in the non-fit condition (vs. fit condition), we aimed to explore on which part of the information participants fixate more: on pros, cons, or both (Hypothesis 2). To test Hypothesis 2, we ran a repeated measure test of variance with the regulatory fit/non-fit condition as a factor. The analysis, showed a non-significant difference between participants’ average fixations on pros or cons, *F*(2,180) = 2.78, *p* = .10. We found a non-significant influence of the interaction between fit/non-fit condition and average fixations on pros or cons, *F*(2,180) = 3.38, *p* = .07. At the same time, the non-fit experience significantly influenced participants tendency to fixate longer on both pros and cons, *F*(2,180) = 7.04, *p* < .01 η^2^_partial_ = .04. Since, we observed that non-fit condition enhances participants’ thorough information processing for both parts of the information: pros & cons, we used the average fixation duration on pros & cons in subsequent analyses.

We ran a MANOVA analysis to compare the total fixation duration (TFD) between fit/non-fit conditions for pros, cons, and information about the vaccine, exploring whether participants motivation to explore more details in the provided information depends on fit/non-fit conditions. Results were in the predicted direction, suggesting that in the non-fit condition participants overall total duration fixation was larger (*M*_non-fit_ = 101.35 sec, *M*_fit_ = 88.85sec) than in the fit condition but the difference did not reach significant levels, *F*(3, 177) = 1.29, *p* = .28.

### Regulatory non-fit and attitude change

**Fig 3:**
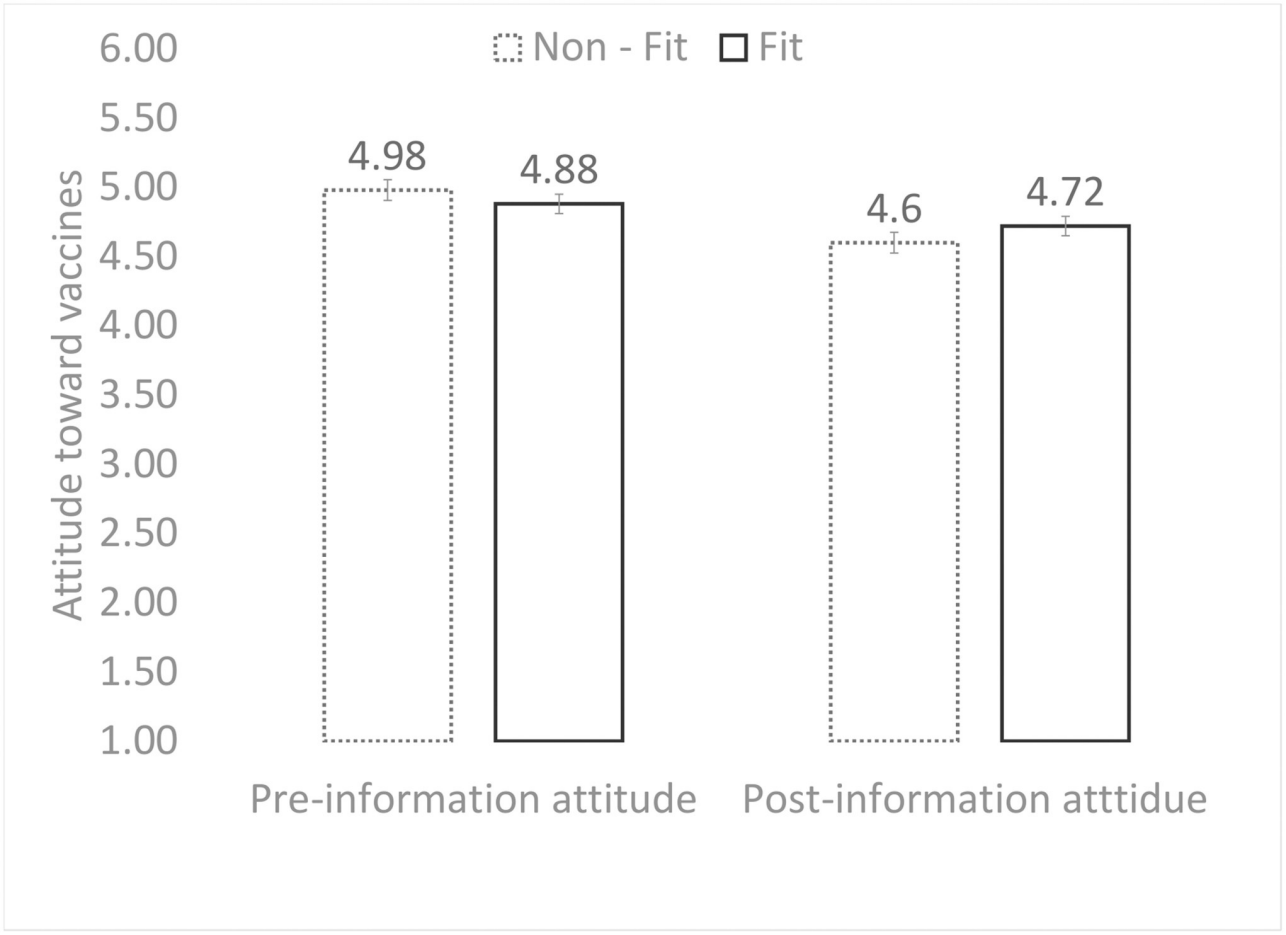
Attitude change as a function of regulatory fit and non-fit experiences (a repeated measure design).

We ran a one-way analysis of variance, to check whether pre-information attitudes differed across the fit and non-fit conditions. There was no difference between participants who were assigned to the fit versus the non-fit condition, *F*(195, 1) = 0.74, *p* = .39. To test for attitude changes toward vaccines (Hypothesis 3), we conducted a 2 x 2 analysis of variance (regulatory condition: non-fit, fit) by the time of attitude assessment (pre- and post-), with repeated measures on the latter factor. As shown in Fig 3, positive attitudes toward vaccination were significantly reduced across both the regulatory fit/non-fit conditions, *F*(194, 1) = 47.69, *p* < .001, η_p_^2^ = .20. More importantly, the interaction was significant, *F*(194, 1) = 6.98, *p* = .01, η_p_^2^ = .03, indicating that, as hypothesized, attitude change (reduction) was stronger in the non-fit condition than in the fit condition. The interaction remained significant when covariates (such as having glasses/contact lenses and past experiences with vaccination) were included in the analysis, *F*(192, 1) = 6.78, *p* = .01, η_p_^2^ = .03. Hypothesis 3 was supported.

### Motivation to process information (Eye-tracking data) and attitude change

**Fig 4:**
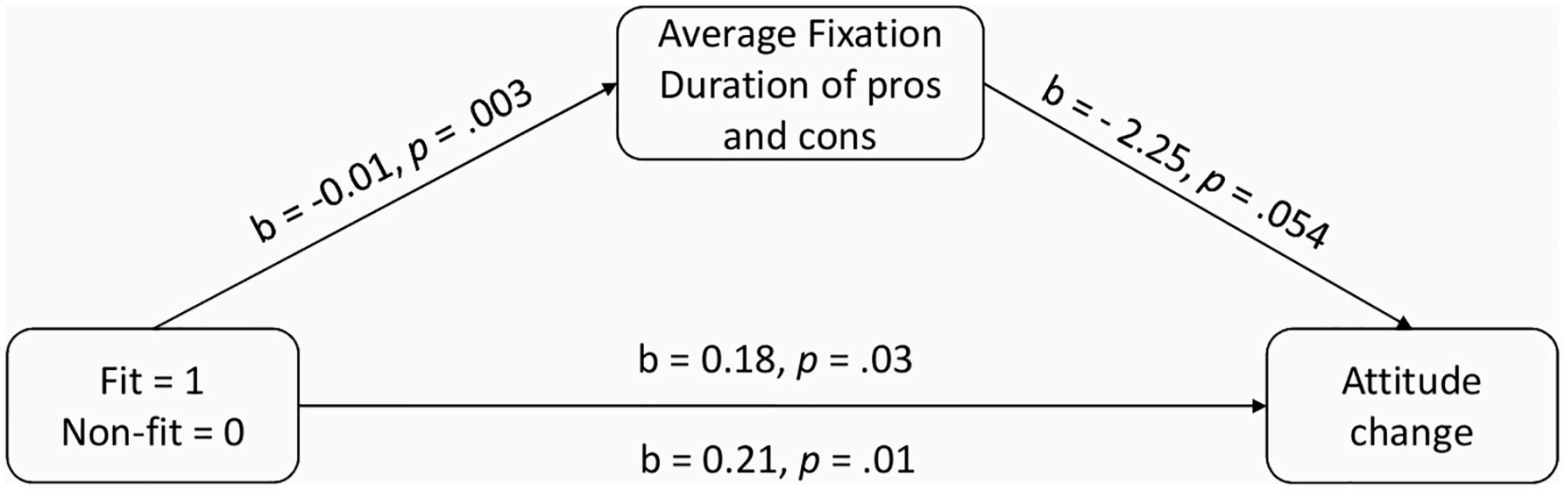
Indirect effect of the non-fit condition on attitude change via average fixation duration (AFD) on pros and cons. For attitude change, lower number means more attitude change.

We examined whether a thorough reading of pros or cons explains the relation between regulatory non-fit and attitude change (Hypothesis 4). We added AFD for pros & cons to the mediation Model 4 (in PROCESS procedure) and ran a bootstrapping analysis with bootstrap samples = 10,000 [34]. Attitude change (post-attitude minus pre-attitude) was included as the dependent variable (for a similar approach see [2]). The indirect effect was significant, R^2^ = .05, *F* (2, 193) = 5.41, *p* <.01, b = 0.03 95%CI [0.01, 0.08], indicating that AFD (average duration fixation) of pros and cons partially mediated the relation between the non-fit experience and attitude change (for all of the statistics, see Fig 4). This analysis suggests that the regulatory non-fit experience motivated participants to read the pros and cons more thoroughly. The more thoroughly regulatory non-fit individuals invested in reading, the more they reduced their initial positive attitude. Hypothesis 4 was supported. To ensure that this effect was not driven by participants’ average duration fixation only on pros or only on cons, we ran additional analysis inserting AFD of each cons and pros as mediators in Model 4. Neither AFD of pros nor AFD of cons explained participants’ attitude change.

### Regulatory non-fit and decision making

A total of 60% of participants chose to get the (fictitious) vaccine despite being given evidence that the vaccine was more likely to cause side effects than protect them. We ran a logistic regression to test Hypothesis 5, predicting that participants will be less likely to vaccinate in the non-fit condition. The results reveal a non-significant difference (*b* = -0.12, *p* = .69). Hypothesis 5 was not supported.

To test Hypothesis 6, we first ran a logistic regression exploring the relationship between attitude change and choice and found significant relationships, 2LL = 253.73, Cox & Snell R^2^ = .05, b = 0.87, *p* <.01, OR adj = 2.38 [1.33, 4.26], meaning that a reduction of attitude in 1 unit makes it 2.4 times more likely that participants reject harmful vaccination.

**Fig 5:**
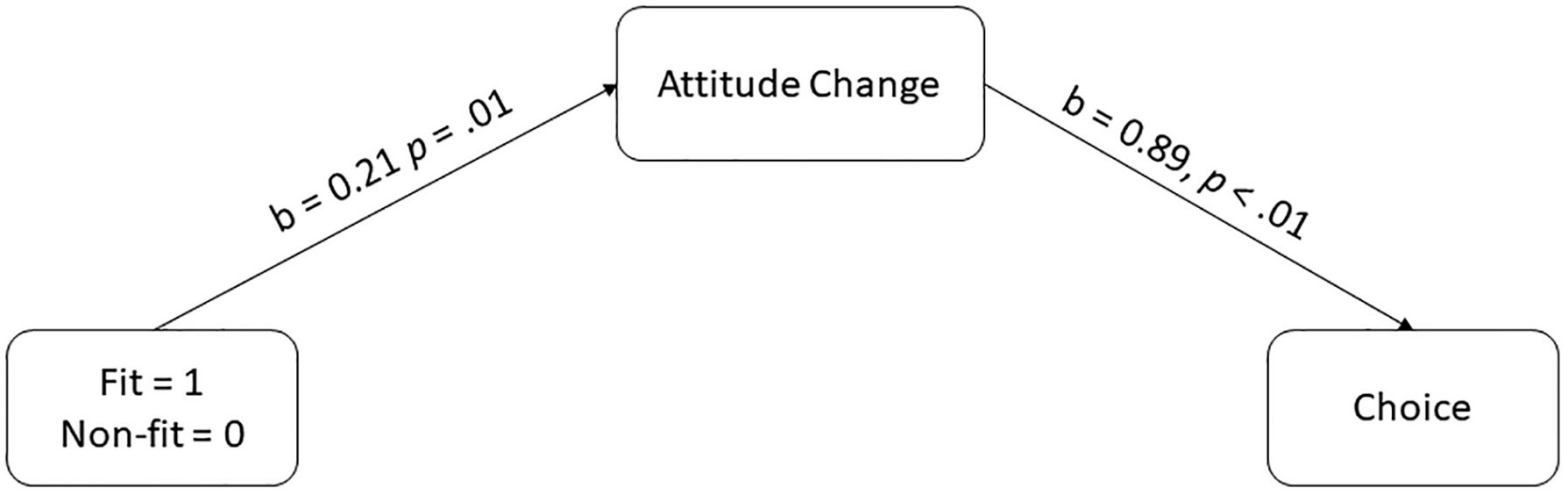
Indirect effect of regulatory fit and non-fit on choices via attitude change (choice variable is coded 0 = reject; 1 = accept).

We then tested our prediction that non-fit might influence choice via reducing initial positive attitudes, and thus helps individuals make better decisions. We explored an indirect effect of attitude change on the relation between regulatory fit/non-fit and participants’ choice, following the recommendations of Preacher and Hayes [35]. We used Model 4 of the PROCESS procedure with bootstrap samples = 10,000 [34]. We found that the indirect effect between non-fit/fit conditions and choice reached a significant level, 2LL = 253.71, Cox & Snell R^2^ = .05, *b* = 0.19 95%CI [0.05, 0.42], supporting Hypothesis 5 (for all of the statistics, see Fig 5). The direct effect and total effect were not significant, suggesting that regulatory non-fit only indirectly influenced participants’ decisions [34, *p*90]. According to previous research in statistics, an indirect effect can occur even if the relation between the independent and dependent variables are not statistically significant [36–39] as in the current case. This analysis suggests that the regulatory non-fit experience reduces the intensity of the initial positive attitude towards vaccines, and that the resulted attitudes influence individuals’ choices to reject the harmful vaccine. Hypothesis 6 was supported. For additional analysis for choice and information processing see S2 Text, S1 Fig.

### Self-reported experiences

Participants did not differ in their perceptions of the vaccine information by fit/non-fit condition, except for their perceptions of how dangerous the fever was, rated on a 7-point Likert scale, *F*(195, 1), *p* = .01, η_p_^2^ = .04. In the fit condition, participants thought that the fever was more dangerous (*M* = 4.24, *SD* = .90) than participants in the non-fit condition (*M* = 3.88, *SD* = .99). However, and importantly, this item was not associated with attitude change (r^2^ = .01, b = 0.12, *p* = .34), AFD (r^2^ = .01, b = -2.72, *p* = .20). Not surprisingly, all participants across conditions (i.e., there was no fit *vs*. non-fit difference), participants found pro-attitudinal (pros) arguments (*M* = 3.82, *SD* = 0.78) more convincing than counter-attitudinal (cons) arguments (*M* = 2.94, *SD* = 1.01), as a pair-sample t-test indicated, *t*(195) = 10.09, *p* <.001 95% CI [0.71, 1.05].

**Fig 6:**
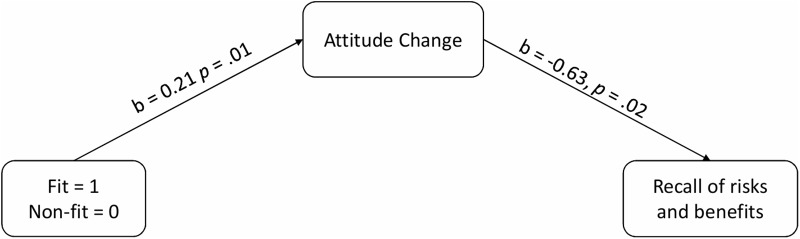
Indirect effect of regulatory fit and non-fit on recall of risks and benefits via attitude change (recall coded as 0 = at least one mistake; 1 = no mistakes).

More importantly, as hypothesized, a logistic regression analysis indicated that the reduction of initial positive attitudes was associated with participants’ accurate recall of the statistics that described risks of the (fictitious) harmful vaccine, 2LL = 263.62, Cox & Snell *R*^2^ = .03, *b* = -0.63, *p* = .02. At the next step, we added attitude change as a mediator in Model 4 (in PROCESS procedure) and ran a bootstrapping analysis with bootstrap samples = 10,000 [34], to test Hypothesis 8. As above, the analysis indicated that while there is no direct effect (Hypothesis 7 was not supported), there is the indirect effect between regulatory non-fit and accurate recall of risks and benefits via attitude change, 2LL = 263.61, Cox & Snell *R*^2^ = .03, *b* = -0.14 95%CI [-0.35, -0.02] (for all of the statistics, see Fig 6). The regulatory non-fit experience reduced the intensity of the initial positive attitude. Participants’ reduced attitudes influenced their accuracy in the recall of risks and benefits of the fictitious harmful vaccine.

## General discussion

This study is the first to use eye-tracking measures to provide empirical evidence that regulatory non-fit influences attitude change via increased information processing. We found that participants fixated on words for a longer time in the non-fit condition (AFD) than in the fit condition’ highlighting their motivation to invest more cognitive efforts in processing information. This finding is consistent with the theoretical assumptions of Regulatory Fit theory. The theory states that when experiencing regulatory non-fit, individuals have a motivational disruption [9,16] that, in turn, makes them invest more cognitive efforts in decision-making. In other words, non-fit makes participants feel wrong and not take anything for granted. This is the first study to observe with eye-tracking the theoretically-predicted effect of the motivational disruption caused by regulatory non-fit (*vs*. fit) on individuals’ willingness to process information more thoroughly.

### Regulatory non-fit and attitude change

We found that regulatory non-fit reduces the intensity of an initially strong positive attitude. Our experiment highlighted that regulatory non-fit can de-intensify not only negative [2] but also positive attitudes. In addition, we uncovered the relation between the message content, fit or non-fit experiences, and attitude change. While previous research coupled non-fit experience and counter-attitudinal messages, in this study, we provided both pro- and counter-attitudinal arguments. The purpose was to test whether pro- or counter-attitudinal information, or both, influence attitude change.

We found that participants processed both the pros and cons of vaccination more thoroughly if they experienced non-fit rather than fit. A mediation analysis did not reveal a separate effect of participants’ processing pros or cons on attitude change. Instead, the relation between regulatory non-fit and attitude change was explained by how thoroughly participants processed both the pros and cons of vaccination. This observation is the first to demonstrate what influences attitude change in the regulatory non-fit condition. Furthermore, this finding suggests that more than one mechanism of attitude change was likely at play.

First, thorough reading of the counter-attitudinal information could contribute to attitude change. The assimilation of counter-attitudinal information could result in de-intensification of initial positive attitudes by reducing the consistency of participants’ initial beliefs [40, 41]. Second, participants’ increased attention to pro-attitudinal information could also help them to learn new facts about vaccination that, in turn, increase the complexity of their beliefs about vaccination [1]. For example, two individuals might have positive attitudes toward vaccination. However, one person has only one dimension underlining his/her belief, such as vaccines could protect health whereas the other person’s belief includes multiple dimensions, such as vaccines keep you healthy, they help protect other people from falling ill, and they are often safe and effective. It is possible that the first individual with less complex beliefs could have stronger attitudes toward vaccination than the second individual with more complex beliefs [42]. Thus, learning pro-attitudinal information could also contribute to attitude change by increasing the complexity of participants’ initial beliefs.

It is likely that in our research both mechanisms were at play. Our design does not allow us to test which of them is more important for attitude change in the regulatory non-fit condition. Further research is needed to empirically test how these mechanisms of attitude change unfold in the regulatory non-fit condition, and when one or the other has the stronger impact on attitude change.

Overall, our findings advance the theory of regulatory non-fit by suggesting that non-fit could reduce the intensity of not only negative attitudes but also positive attitudes. Furthermore, we showed that regulatory non-fit could change attitudes when coupled with both counter-attitudinal and pro-attitudinal information. Additionally our results contribute to the literature on persuasion [1, 28] by suggesting that regulatory non-fit could be utilized as a means for opinion change.

### Regulatory non-fit and decisions

While further research is needed to explore the direct effect of regulatory non-fit on individuals’ decisions, we found that participants who reduced their initial positive attitudes toward vaccination were more likely to correctly recall the risks of the harmful vaccination, and then reject the vaccination. Importantly, our analysis of the indirect effect showed that regulatory non-fit was a precondition for the influence of attitude reduction on participants’ choices. Notably, in both analysis, we found significant but relatively small associations between “choice”/ “recall” measures and attitude change.

While present results call for further research, our findings offer intriguing insight on how to facilitate informed choice. Using regulatory non-fit could be helpful if individuals deal with choices toward which they have strong positive or negative initial attitudes that could bias their choices.

These results add to the literature on information processing [43–44]. In the previous research, it was proposed that regulatory non-fit could boost information processing [2, 17, 18]. However, until this study, there were only indirect observations of information processing in regulatory fit and non-fit conditions. Our results are the first to observe directly (via eye-tracking procedure) that experiencing regulatory non-fit individuals indeed processed information more thoroughly than when they experienced regulatory fit. These findings are generally consistent with the previous work that showed how incongruent (in various domains) messages facilitated an elaborative information processing [19–21]. Our research suggests that regulatory non-fit could be a means for creating incongruence in communication that, in turn, facilitates information processing.

Our finding is a novel contribution to an individualized message framing in healthcare communication. Multiple studies showed that individuals tend to be more motivated to follow a message recommendation if it is tailored to match their individual characteristics, for example motivational orientations [15] or personality traits [45–46]. Our study is among the first to demonstrate the advantages of strategically mismatching a message and an individual’s characteristics. Specifically, non-fitting a message could be helpful when people’s baseline attitudes are not supported by statistical evidence [47].

In practice, consultants could frame information to create a regulatory non-fit experience and provide either counter-attitudinal arguments or both pro- and counter-attitudinal arguments. Promotion-focused individuals, for example, might be more motivated to read the information about an option they strongly like and currently think “I know all about it,” if the information they receive emphasizes losses they could avoid by not choosing that option (i.e., a non-fit vigilant message). In this case, the message will make individuals feel “wrong” and, as a result, will increase individuals’ willingness to attend to the information more thoroughly. Thorough processing of both pro- and counter-attitudinal arguments could de-intensify or de-bias prior attitudes. Individuals with less intense attitudes might be more open toward considering both the benefits and the costs of a discussed option rather than follow their attention selectivity bias and concentrate on just the information that confirms their previous attitudes.

We recognize that it would be relatively rare that a positive attitude toward vaccination would need to be reduced, but we chose to use a vaccination example as it allowed us to experiment with naturally occurring attitudes that students on campus have toward a medical procedure. Nonetheless, in the real-world of medical decision making, positive attitudes might be problematic and need to be reduced because they, for example, impede individuals from considering risks of invasive procedures that cause complications but have little benefits for patients.

### Limitations and suggestions for future research

The present research has several limitations that, in turn, may offer some fruitful suggestions for future research. First, a non-fit experience is not restricted to the case where the motivational orientations are regulatory focus orientations [3, 4]. It can result from various motivational orientations, such as regulatory mode [48], or construal levels [49]. It would be interesting in future research to test regulatory non-fit effects on de-intensifying positive or negative attitudes with different motivational orientations. We expect that the same basic effects would be found.

Second, in our study, participants read general information about vaccination. Right after reading it, they received the specific information about a (fictitious) harmful vaccine. This approach prevents us from exploring the independent effects of general and specific information on attitude change. We believe that reading about the potential risks of a particular (fictitious) harmful vaccine could have contributed to a reduction of positive attitudes toward vaccination in general in both the fit and non-fit conditions, which would weaken the “non-fit versus fit effect” on participants’ attitudes and choices. Further research should address this limitation and test the effect of these two types of information separately. Notably, despite this limitation, we still found a “non-fit versus fit effect” on the reduction of the initial positive attitude.

Third, participants made a decision evaluating a hypothetical vignette in a behavioral laboratory. At this stage of our research, it would not be ethical or feasible to manipulate information framing in a real-life context. We aimed first to find evidence that the proposed non-fit intervention indeed helped individuals to make informed decisions. Thus, we developed a hypothetical vignette that, unlike most real decisions, had a “good” and a “bad” choice. While participants’ engagement and choices might be influenced by artificial settings of laboratory experiments, this design allowed us to explore whether the non-fit intervention helps individuals make a choice that would lead to a better medical outcome. The difference that we observed between fit and non-fit conditions suggests that there might be an interesting phenomenon to explore further in filed experiments. The next step would be to evaluate regulatory non-fit in clinical settings, in which patients’ initial beliefs might interfere with their willingness to evaluate medical evidence thoroughly.

Fourth, effect sizes of statistical analysis in this study have a relatively small magnitude. That could be a factor of a) participants’ levels of engagement in the laboratory settings, b) participants’ exposure to both pro- and counter- attitudinal information c) participants’ not-so-extreme attitudes toward vaccination. Thus, we propose to run further studies that allow observing participants’ natural behavior; participants’ attitude change when they encounter only counter-attitudinal information; and participants’ attitude change toward a topic that might induce stronger attitudes (e.g., euthanasia).

### Conclusion

The present findings align with a growing body of research on regulatory non-fit effects, and suggest that framing message information in a non-fit manner with individuals motivational orientation can encourage more thorough information processing and reduce the impact of prior attitudinal biases on medical choices.

## Supporting information

S1 Text Materials & measures. (DOCX)

S2 Text Processing of pros and cons and choice. (DOCX)

S1 Fig Time participants spend reading pros and cons. (TIF)

S1 Table Correlation table. (DOCX)

## References

[1] Eagly AH, and Chaiken S, The handbook of social psychology, in *Attitude structure and function*, Gilbert, & Lindzey, Editors. 1998, McGraw-Hill: New York, NY p. 269–322.

[2] Fridman I, Scherr KA, Glare PA, Higgins ET. Using a Non-Fit Message Helps to De-Intensify Negative Reactions to Tough Advice. Personality and Social Psychology Bulletin. 2016 8;42(8):1025–44. 10.1177/0146167216649931 27341845 PMC5610136

[3] Higgins ET. Beyond pleasure and pain. American psychologist. 1997 12;52(12):1280–1300. 9414606 10.1037//0003-066x.52.12.1280

[4] Higgins ET. Promotion and prevention: Regulatory focus as a motivational principle In Advances in experimental social psychology 1998 Jan 1 (Vol. 30, pp. 1–46). Academic Press.

[5] Higgins ET, Silberman I. Development of regulatory focus: Promotion and prevention as ways of living In Heckhausen J. & Dweck C. S. (Eds.), *Motivation and self-regulation across the life span* (pp. 78–113). New York, NY, US: Cambridge University Press.

[6] Halvorson HG. Focus: Use different ways of seeing the world for success and influence. 2013. Penguin.

[7] Lanaj K, Chang CH, Johnson RE. Regulatory focus and work-related outcomes: a review and meta-analysis. Psychological bulletin. 2012 9;138(5):998–1034. 10.1037/a0027723 22468880

[8] Higgins ET, Friedman RS, Harlow RE, Idson LC, Ayduk ON, Taylor A. Achievement orientations from subjective histories of success: Promotion pride versus prevention pride. European Journal of Social Psychology. 2001 1 1;31(1):3–23.

[9] Higgins ET. Value from hedonic experience and engagement. Psychological review. 2006 7;113(3):439–460. 10.1037/0033-295X.113.3.439 16802877

[10] Higgins ET. Promotion and prevention: How “0” can create dual motivational forces. Dual-process theories of the social mind. 2014 5 1:423–436.

[11] Cesario J, Grant H, Higgins ET. Regulatory fit and persuasion: Transfer from" feeling right.". Journal of personality and social psychology. 2004 3;86(3):388–404. 10.1037/0022-3514.86.3.388 15008644

[12] Lee AY, Aaker JL. Bringing the frame into focus: the influence of regulatory fit on processing fluency and persuasion. Journal of personality and social psychology. 2004 2;86(2):205–218. 10.1037/0022-3514.86.2.205 14769079

[13] Higgins ET, Idson LC, Freitas AL, Spiegel S, Molden DC. Transfer of value from fit. Journal of personality and social psychology. 2003 6;84(6):1140–1153. 12793581 10.1037/0022-3514.84.6.1140

[14] Idson LC, Liberman N, Higgins ET. Imagining how you’d feel: The role of motivational experiences from regulatory fit. Personality and Social Psychology Bulletin. 2004 7;30(7):926–937. 10.1177/0146167204264334 15200698

[15] Ludolph R, Schulz PJ. Does regulatory fit lead to more effective health communication? A systematic review. Social science & medicine. 2015 3 1;128:142–150. 25617673 10.1016/j.socscimed.2015.01.021

[16] Higgins ET. Making a good decision: value from fit. American psychologist. 2000 11;55(11):1217–1230. 11280936

[17] Koenig AM, Cesario J, Molden DC, Kosloff S, Higgins ET. Incidental experiences of regulatory fit and the processing of persuasive appeals. Personality and Social Psychology Bulletin. 2009 10;35(10):1342–1355. 10.1177/0146167209339076 19571272

[18] Vaughn LA O’Rourke T, Schwartz S, Malik J, Petkova Z, Trudeau L. When two wrongs can make a right: Regulatory nonfit, bias, and correction of judgments. Journal of Experimental Social Psychology. 2006 9 1;42(5):654–661.

[19] Maheswaran D, Chaiken S. Promoting systematic processing in low-motivation settings: Effect of incongruent information on processing and judgment. Journal of personality and social psychology. 1991 7;61(1):13–25. 1890583 10.1037//0022-3514.61.1.13

[20] Alter AL, Oppenheimer DM, Epley N, Eyre RN. Overcoming intuition: metacognitive difficulty activates analytic reasoning. Journal of Experimental Psychology: General. 2007 11;136(4):569–576. 17999571 10.1037/0096-3445.136.4.569

[21] Hernandez J. Disfluency disrupts the confirmation bias: when changing the font changes your mind.

[22] Ferrer RA, Stanley JT, Graff K, Klein WM, Goodman N, Nelson WL, Salazar S. The Effect of Emotion on Visual Attention to Information and Decision Making in the Context of Informed Consent Process for Clinical Trials. Journal of Behavioral Decision Making. 2016 4 7;29(2–3):245–253.

[23] Just MA, Carpenter PA. A theory of reading: From eye fixations to comprehension. Psychological review. 1980 7;87(4):329–354. 7413885

[24] Kuo FY, Hsu CW, Day RF. An exploratory study of cognitive effort involved in decision under Framing—an application of the eye-tracking technology. Decision Support Systems. 2009 12 1;48(1):81–91.

[25] Rayner K, Duffy SA. Lexical complexity and fixation times in reading: Effects of word frequency, verb complexity, and lexical ambiguity. Memory & cognition. 1986 5 1;14(3):191–201. 3736392 10.3758/bf03197692

[26] Zola D. The effect of redundancy on the perception of words in reading. Center or the Study of Reading Technical Report; no. 216. 1981.

[27] Petty RE, Cacioppo JT. Issue involvement can increase or decrease persuasion by enhancing message-relevant cognitive responses. Journal of personality and social psychology. 1979 10;37(10):1915–1926.

[28] Tsai CI, Klayman J, Hastie R. Effects of amount of information on judgment accuracy and confidence. Organizational Behavior and Human Decision Processes. 2008 11 1;107(2):97–105.

[29] Festinger L. A theory of cognitive dissonance. Stanford university press; 1962.

[30] Rayner K. Eye movements in reading and information processing: 20 years of research. Psychological bulletin. 1998 11;124(3):372–422. 9849112 10.1037/0033-2909.124.3.372

[31] Gliem JA, Gliem RR. Calculating, interpreting, and reporting Cronbach’s alpha reliability coefficient for Likert-type scales. Midwest Research-to-Practice Conference in Adult, Continuing, and Community Education.

[32] Streiner DL. Starting at the beginning: an introduction to coefficient alpha and internal consistency. Journal of personality assessment. 2003 2 1;80(1):99–103. 10.1207/S15327752JPA8001_18 12584072

[33] Tavakol M, Dennick R. Making sense of Cronbach’s alpha. International journal of medical education. 2011;2:53–55. 10.5116/ijme.4dfb.8dfd 28029643 PMC4205511

[34] Hayes AF. Introduction to mediation, moderation, and conditional process analysis: A regression-based approach. Guilford Publications; 2017 12 13.

[35] Preacher KJ, Hayes AF. SPSS and SAS procedures for estimating indirect effects in simple mediation models. Behavior research methods, instruments, & computers. 2004 11 1;36(4):717–731. 10.3758/bf03206553 15641418

[36] Collins LM, Graham JJ, Flaherty BP. An alternative framework for defining mediation. Multivariate Behavioral Research. 1998 4 1;33(2):295–312. 10.1207/s15327906mbr3302_5 26771887

[37] Judd CM, Kenny DA. Process analysis: Estimating mediation in treatment evaluations. Evaluation review. 1981 10;5(5):602–619.

[38] MacKinnon DP, Krull JL, Lockwood CM. Equivalence of the mediation, confounding and suppression effect. Prevention science. 2000 12 1;1(4):173–181. 11523746 10.1023/a:1026595011371 PMC2819361

[39] Shrout PE, Bolger N. Mediation in experimental and nonexperimental studies: new procedures and recommendations. Psychological methods. 2002 12;7(4):422–455. 12530702

[40] Chaiken S, Baldwin MW. Affective-cognitive consistency and the effect of salient behavioral information on the self-perception of attitudes. Journal of Personality and Social Psychology. 1981 7;41(1):1–12.

[41] Chaiken S, Yates S. Affective-cognitive consistency and thought-induced attitude polarization. Journal of Personality and Social Psychology. 1985 12;49(6):1470–1481. 4087140 10.1037//0022-3514.49.6.1470

[42] Linville PW. The complexity–extremity effect and age-based stereotyping. Journal of personality and social psychology. 1982 2;42(2):193–211.

[43] Chaiken S. Heuristic versus systematic information processing and the use of source versus message cues in persuasion. Journal of personality and social psychology. 1980 11;39(5):752–766.

[44] Petty RE, Cacioppo JT. The elaboration likelihood model of persuasion In Communication and persuasion 1986 (pp. 1–24). Springer New York.

[45] Hirsh JB, Kang SK, Bodenhausen GV. Personalized persuasion: Tailoring persuasive appeals to recipients’ personality traits. Psychological science. 2012 6;23(6):578–581. 10.1177/0956797611436349 22547658

[46] Noar SM, Benac CN, Harris MS. Does tailoring matter? Meta-analytic review of tailored print health behavior change interventions. Psychological bulletin. 2007 7;133(4):673–693. 10.1037/0033-2909.133.4.673 17592961

[47] Fridman I, & Higgins ET, *Regulatory Focus and Regulatory Fit in Health Messaging* Oxford Research Encyclopedia of Communication. 2017 Oxford Press

[48] Avnet T, Higgins ET. Locomotion, assessment, and regulatory fit: Value transfer from “how” to “what”. Journal of Experimental Social Psychology. 2003 9 1;39(5):525–530.

[49] Trope Y, Liberman N. Temporal construal. Psychological review. 2003 7;110(3):403–421. 12885109 10.1037/0033-295x.110.3.403

