# Supplementary material for: Eye-tracking evidence shows that non-fit messaging impacts attention, attitudes and choice
Source: PLoS One. 2018 Oct 26;13(10):e0205993. doi: 10.1371/journal.pone.0205993 (PMC6203368; doi:10.1371/journal.pone.0205993)
Supplement: S1 Text — (DOCX) [file pone.0205993.s001.docx]

**Materials & Measures**

**Promotion and Prevention Priming**

|  |
| --- |

**Promotion.** Please write about a time in the past when:

1. You felt you made progress toward being successful in life
2. Compared to most people, you were able to get what you wanted out of life
3. Trying to achieve something important to you, you performed as well as you ideally would have liked to

**Prevention.** Please write about a time in the past when:

1. Being careful enough avoided getting you into trouble.
2. Growing up, you stopped yourself from acting in a way that your parents would have considered objectionable (unacceptable).
3. Growing up, you were careful not to get on your parents’ nerves.

Attitude Measure

1. Vaccines often lead to positive outcomes (help people stay healthy).
2. Vaccines often lead to negative outcomes (cause diseases or complications).
3. Vaccines are effective in preventing diseases.
4. Vaccines help people to enhance their health.
5. Whenever it is possible, I choose to avoid getting vaccinated.
6. Whenever it is possible, I choose to receive vaccination.
