# Supplementary material for: Eye-tracking evidence shows that non-fit messaging impacts attention, attitudes and choice
Source: PLoS One. 2018 Oct 26;13(10):e0205993. doi: 10.1371/journal.pone.0205993 (PMC6203368; doi:10.1371/journal.pone.0205993)
Supplement: S2 Text — (DOCX) [file pone.0205993.s002.docx]

**Processing of Pros and Cons and Choice**

**Results**

To check whether increased information processing influences participants’ choices, we ran a logistic regression separately with AFD and TFD. Neither AFD on pros, cons or both were associated with participants’ choices. At the next step, we explored whether total duration fixations (TFD) on pros, cons or both were associated with participants’ choices. To do so, we created two variables: (TFD) cons divided by sum pros and cons; and (TFD) pros divided by the sum of pros and cons. The logistic regression showed that participants who spent longer fixating on cons were more likely to choose the vaccination, 2LL = 242.05, Cox & Snell R^2^ = .04, b = 4.15, *p* =.007, OR adj = 63.44 [3.14, 1280.30]. Those participants who spent more time fixating on pros were more likely to reject the vaccination, 2LL = 242.09, Cox & Snell R^2^ = .04, b = -4.06, *p* =.01, OR adj = 0.17 [0.001, 0.40].

**S1 Fig 1: Time participants spend reading pros and cons**

**Discussion**

We found that the total time participants spent reading pros or cons influenced their choices. Specifically, those who read cons longer were more likely to choose the harmful fictitious vaccination, while those who read pros longer were less likely to choose the vaccination. These findings support previous pioneering work on attention bias [1] showing that attention plays an important role in construction of participants’ preferences and values.

Different strategies of participants reading might be also a possible explanation of the observed results (S3 Fig 1). Those participants who skimmed the text spent equal time on both pros and cons. Since they were skimming, they paid less attention to the information; as a result, they chose to take the harmful vaccination. At the same time, participants who spent more time reading the information on the left side of the page (pros), also spent less time reading the right side of the page (cons) because they were likely to notice that the arguments in the cons section covered the same domains as the arguments in the pros section. Thus, participants who read half of the page carefully did not need to spend a long time reading the other side of the page. In future research, we propose to investigate alternative explanations of this phenomenon. To better distinguish between participants’ attention toward pros and cons, the study design should allow participants to choose whether to read pros, cons, or both rather than receive both types of arguments on the same page.

**Reference**

1. Ashby NJ, Dickert S, Glöckner A. Focusing on what you own: Biased information uptake due to ownership. Judgment and Decision Making. 2012 May 1;7(3):254.
