# Supplementary figures and images for: Eye-tracking evidence shows that non-fit messaging impacts attention, attitudes and choice
Source: PLoS One. 2018 Oct 26;13(10):e0205993. doi: 10.1371/journal.pone.0205993 (PMC6203368; doi:10.1371/journal.pone.0205993)

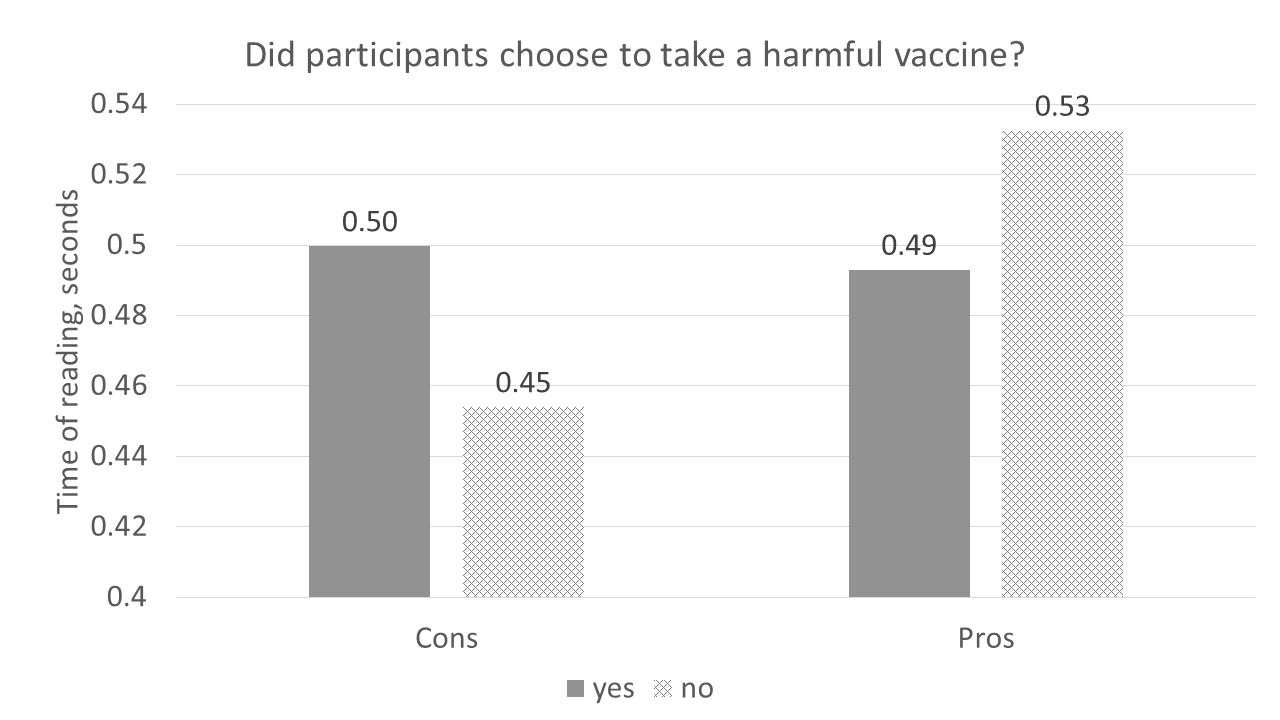

Supplement: S1 Fig — (TIF) [file pone.0205993.s003.TIF]
