# Supplementary material for: Eye-tracking evidence shows that non-fit messaging impacts attention, attitudes and choice
Source: PLoS One. 2018 Oct 26;13(10):e0205993. doi: 10.1371/journal.pone.0205993 (PMC6203368; doi:10.1371/journal.pone.0205993)
Supplement: S1 Table — (DOCX) [file pone.0205993.s004.docx]

**Correlation Table**

**Descriptive statistic**

Table 1 summarizes correlational and descriptive statistics. This analysis reveals that regulatory non-fit is associated with both attitude change and average fixation duration. Furthermore, a stronger positive post-information attitude was associated with participants’ decision to take the harmful vaccine, while attitude change that reduced the positive attitude was associated with the decision not to take the vaccine.

*S1. Table 1:* Descriptive statistics and correlations for primary DV and IV
